# Linking telomere dynamics to evolution, life history and environmental change: perspectives, predictions and problems

**DOI:** 10.1007/s10522-023-10081-8

**Published:** 2024-01-22

**Authors:** Pat Monaghan

**Affiliations:** School of Biodiversity, One Health and Veterinary Medicine, Graham Kerr Building University of Glasgow, Glasgow, G12 8QQ UK

**Keywords:** Ecology, Conservation, Environment, Fitness, Comparative, Interdisciplinary

## Abstract

This perspectives paper considers the value of studying telomere biology outside of a biomedical context. I provide illustrative examples of the kinds of questions that evolutionary ecologists have addressed in studies of telomere dynamics in non-model species, primarily metazoan animals, and what this can contribute to our understanding of their evolution, life histories and health. I also discuss why the predicted relationships between telomere dynamics and life history traits, based on the detailed cellular studies in humans and model organisms, are not always found in studies in other species.

## Introduction

Since Alexey Olovnikov recognised that the shortening of chromosome ends in eukaryotes during DNA replication could limit the replicative potential of cells (Olovnikov 1973), enormous strides have been made in our understanding of the cellular processes responsible for telomere maintenance, regulation and attrition (Shay and Wright 2019). By far the majority of this work has been on human and model organism cells in a laboratory setting. The field has been driven forward by recognition of the importance of telomere dynamics in human health, disease and age-related deterioration (Lopez-Otin et al. 2023). It has been demonstrated that telomere attrition and dysfunction are both a cause and a consequence of cellular and molecular ageing. Telomere loss is considered by the biomedical and ageing research community to be a primary hallmark of ageing, and pathways that link telomere dysfunction to age related disease and to other established hallmarks of ageing have been elucidated (Chakravarti et al. 2021; Lopez-Otin et al. 2023). It is only relatively recently that co-operation across disciplinary divides has resulted in a broadening of telomere biology to encompass how this highly conserved process has been shaped by evolution to suit the requirements of species that differ in their habitats, life histories, body sizes, longevity, regenerative capacity and environmental challenges (Monaghan et al. 2022). In this perspective piece, I discuss how evolutionary ecologists have studied telomere dynamics in diverse species to examine the variation within and among individuals, populations and species, and what this can contribute to our understanding of their evolution, life histories and health.

There has been a rapid increase in the number of published papers that involve studies of telomere dynamics in non-model animal species in the wild since the late 1990s (Salmon and Burraco 2022). As outlined in a recent over view (Tobler et al. 2022), this research is carried out primarily by organismal-level biologists and evolutionary ecologists, with interests in understanding processes that drive the evolution and diversity of species and individual life histories. It largely centres on how variation in telomere dynamics is related to variation in organismal level performance, linking telomere length and loss to environmental conditions and key life history traits such as growth, reproductive success and survival, and thereby to Darwinian fitness outcomes. The work involves both observational and experimental work with an increasing diversity of species, carried out in the laboratory and in the field. The predominant use of blood samples in these studies is in part because such samples are much easier to obtain relatively non-invasively, and, in non-mammalian vertebrates, the nucleated red blood cells mean that large amounts of DNA can be obtained from very small samples. Also, when looking at age-related changes in traits, evolutionary ecologists have been much more concerned than biomedical researchers about the biases that can occur due to ‘selective disappearance’ of phenotypes in cross sectional studies; patterns of change with age can appear or be masked by differential survival of individuals with long or short telomeres. Hence the desire to look at within-individual changes where possible, using minimally invasive approaches that enable individuals to be sampled repeatedly and enable species of high conservation concern to be studied (Nussey et al. 2014).

The study of diversity in telomere dynamics is now of considerable interest to a broad church of biologists and interdisciplinary collaboration is increasing, as reflected in recent journal special issues (e.g. see special issues of Philosophical Transactions of the Royal Society *Understanding Diversity in Telomere Dynamics* (Monaghan et al. 2018) and Molecular Ecology *Telomeres in Ecology and Evolution* (Monaghan et al. 2022). The recent examples I give below illustrate the general approach of evolutionary biologists and ecologists studying telomere dynamics and the kinds of questions addressed. I also consider to what extent telomere dynamics might help us identify species and life stages whose welfare is most compromised, and those that are at most risk of being driven to extinction by the current rapid pace of environmental change. The basic biology of telomeres is highly conserved by evolution. Yet, the expected patterns and organismal level outcomes of changes in telomere length, based on the studies by biomedical scientists in humans and model organisms, are not always found. I therefore also discuss why this is so.

## Can telomere length provide information on individual age?

The idea that telomere length might provide a useful measure of the chronological age of individual animals, where actual age is unknown, was put forward in a pioneering paper by Haussman and Vleck (2002). However, while it was clear from their initial studies with zebra finches *Taeniopygia guttata* that telomere length showed a measurable change with age that could be repeatedly measured using DNA from red blood cells, it became clear that, within species, there is generally too much variation in telomere length among individuals of the same chronological age for telomere length to give a sufficiently accurate measure. This variation itself however was recognised as being of great interest (Monaghan and Haussmann 2006). In fact, there can be significant differences in telomere length among populations or ecotypes of the *same* species, as for example in the different reproductive morphs of the common lizard *Zootoca vivipara* (McLennan et al. 2019) in the wild. Laboratory populations of mice have much longer telomeres than their wild counterparts, and such elongation of telomeres in captivity is even seen in the model organism, the budding yeast *Saccharomyces cervisiae*, in which there is also heterogeneity in telomere length among natural strains linked to aspects of mitochondrial metabolism (D’Angiolo et al. 2023). Why telomere elongation occurs in captivity is unclear but is presumably related to the preferential breeding of individuals that tolerate these conditions well.

In a recent meta-analysis of the association between telomere length and age across 98 species of vertebrates, Remot et al. (2022) found an overall negative relationship, but this was weak and varied across the vertebrate classes. While factors such as the method used to measure telomere length, and publication bias, play a role, only in birds did they detect a reasonably strong effect. This is not unexpected. The pattern of change in telomere length with age will vary among species as a consequence of variation in its effects on Dawinian fitness. Epigenetic clocks may prove to be more useful indicators of chronological age (Lu et al. 2023). Studies in humans suggest that changes in DNA methylation and telomere length are not closely related (Marioni et al. 2016), though limited data to date suggest that this is not always so: for example, the expected negative relationship between the change in DNA methylation and telomere loss is present in wild zebra finches in early life (Sheldon et al. 2022). It will be interesting to see if such relationships change across the life course, since the two measures might be differentially influenced by environmental circumstances later in life.

## Is variation in telomere length and/or loss rate related to variation in longevity within and among species?

### Within species

The expected negative relationship between telomere length and mortality risk has been demonstrated at the individual level in a wide range of species. This relationship seems to be particularly evident in endotherms, especially birds, for which there are most data (e.g. Gomes et al. 2011; Wilbourn et al. 2018). However, a negative relationship is not seen in all endotherm species studied so far, and is much less evident in ectotherms than in endotherms, perhaps because there is more variation in the extent to which telomerase is expressed in ectotherm somatic tissues (Olsson et al. 2018). Within the endotherms, an important difference between studies of mammals and birds is that, even when blood samples are used, in birds the DNA generally comes from their nucleated red blood cells but in mammals it is from white blood cells. Particularly in the wild, changes in the composition of the white blood cell population can occur with age, sex, infection status and social circumstances (Nussey et al. 2012). Furthermore, the many sources of variation in natural systems, among individuals, species and environments and the greater exposure of wild animals to infection and stressors, make standardising studies, using white blood cells, in the wild very difficult. A within-individual study in a cohort of captive zebra finches, followed from birth to death and experiencing the same consistent environmental conditions, showed that telomere length at the end of the growth period was the best predictor of longevity (Heidinger et al. 2012). On the other hand, a detailed, long-term, individual-based study of an isolated feral population of Soay sheep *Ovis aries* found no association between leucocyte telomere loss and mortality risk (Froy et al. 2021). This difference might be due to taxon specific effects, to the difference between patterns in white and red blood cells, or to the more variable and challenging environment experienced in the wild where there is more exposure to stressors disease and extrinsic sources of mortality. Interestingly, in the sheep study, differences among individuals in their lifetime average telomere length were linked to longevity, and these differences were found to have a heritable component, suggesting that inherited variation in telomere length is indicative of individual quality in this species.

Telomere loss is more difficult to study than telomere length, since animals need to be re-captured, the elapsed time between samples can be long or short relative to the animal’s lifespan, and certain phenotypes are absent in the older age groups due to their poorer survival. In a detailed study of a long-lived seabird, the common tern *Sterna hirund*o, Vedder et al. (2022) found that there was a very consistent pattern of telomere loss with age among individuals; telomere length on the other hand varied among individuals and again, as in the sheep, this was a highly heritable trait. That some individuals start life with shorter telomere length than others could have important effects on their longevity, since adverse effects of telomere dysfunction would be expected to start at an earlier age in such individuals. Offspring of older age mothers and fathers have been found to have reduced lifespans in many species (Monaghan et al. 2020), which could arise if their offspring inherit shorter telomeres, as has been reported for offspring of old parents of both sexes in birds (Noguera et al. 2018; Marasco et al. 2019). Variation in the age of breeders is therefore an additional source of variation in telomere length.

However, heritability of telomere length is not always found to be high (Dugdale and Richardson 2018). Differences in developmental and growth conditions, and in the age, life stage and elapsed period over which individuals are sampled, will all be important sources in variability, as will the stability and harshness of the environment (Monaghan 2014; Dugdale and Richardson 2018; Entringer et al. 2018).

### Among species

Consistent interspecific differences in telomere length and loss can be considerable even among closely related species, as for example in killifish (Reichard et al. 2022). Consequently several authors have examined the relationships between telomere dynamics and lifespan across species in a quest to find general relationships. Such comparative analyses generally require data from a large number of species in order to have sufficient power to include as co-variates the many differences in species biology and their degree of common ancestry. Standardising the sampling and measurement protocols from different species is very challenging, since there can be substantial differences in the age range, life history stages sampled, the tissue used and the measurement method. There can also be population, cohort and environmental effects. For example, using phylogenetically corrected data on telomeres from cultured fibroblast cells, collected from a range of tissues in over 60 species of mammals and grown in a variety of media, an impressive comparative study was carried out by Gomes et al. (2011). They suggested that the ancestral mammalian system was short telomeres and downregulated somatic telomerase; telomere length was inversely correlated with maximum lifespan, while telomerase expression co-evolved with body size, with large bodied species having less somatic telomerase expression than smaller bodied species, in line with prior findings in rodents (Gorbunova et al. 2007). Gomes et al. also suggested that this ancestral mammalian system initially evolved as an adaptative response to the need to limit cell replicative potential, driven by the increased mutation rate arising from homeothermy. This suggestion is very interesting but has received little subsequent attention. A recent re-analysis of Gomes et al.’s data supported the inverse relationship between telomere length and maximum lifespan (Pepke and Eisenberg 2022), with the resulting curtailment of cell replicative potential being thought to be tumour protective. However, the extent to which data from cells cultured in vitro are truly representative is problematic, since telomere shortening rate appears to be significantly higher in vitro than in vivo (Lai et al. 2018).

In contrast, Tricola et al. found no association between the average species telomere length and maximum lifespan in birds, based on cross-sectional data from red blood cell samples from 19 species of long-lived birds in natural populations (Tricola et al. 2018). However, they did find that telomere loss rate was strongly negatively associated with maximum lifespan across species. Criscuolo et al. (2021) performed a meta-analysis of variation in mean telomere length in chicks and adults of different bird species, and mean change in telomere length in relation to maximum lifespan and a range of other life history traits (body size, growth rate, speed of development pre-and post-natally and reproductive rate). They used data from 53 species, from 13 orders and 29 families. They found little phylogenetic or life history association for telomere length. Telomere loss rate, on the other hand, did show a strong phylogenetic signal, which they suggested was probably related to the tendency for closely related species to have similar life histories. They found that, across the species they examined, telomere loss was slower in long lived species that have slow reproductive and embryonic growth rates. Telomere loss rate has similarly been found to be slower in long-lived than in short-lived mammals and birds (Dantzer and Fletcher 2015). Dobson et al. (2022) also recently reported no association between telomere length and measures of longevity based on 30 species of birds. However, they did find that telomere loss rate was strongly related to body mass independent lifespan, which they suggest is related to pace of life. In another recent analysis based on 57 bird species, it was found that mean early life telomere length was shorter in species with low reproductive rates and long lifespans (Le Pepke et al. 2022); the life stage at which telomere length measurements are made may therefore be very important. Overall, the picture at present suggests that telomere loss rather than length per se is more likely to be related to maximal lifespan across species. It should be noted however, that in many studies by evolutionary ecologists, relative rather than absolute telomere length is used, often due to sample number and sampling difficulties (Nussey et al. 2014). This limits the scope for comparative studies of telomere length.

## Is variation in growth and reproductive rate related to differences in telomere dynamics?

In general, most animals do not grow at their fastest possible rate, and growth rate is optimised by natural selection rather than maximised, due to the costs associated with rapid growth. These costs are often manifest in reduced longevity (Metcalfe and Monaghan 2001, 2003). The most consistent and significant pattern that Criscuolo et al. found in their cross-species comparison mentioned above, was that slow growth and low reproductive rate were associated with lower rates of telomere loss. Experimental acceleration of growth within species has also been shown to increase telomere loss (Monaghan and Ozanne 2018; Salmon et al. 2021). Pre-natal growth conditions are likely to be very important and there seems to be heterogeneity among species in the extent to which telomere length does (Noguera et al. 2016) or does not (Vedder et al. 2017) vary with hatching order in avian broods, most probably related to the degree of variation in egg composition, which will affect variation in pre-natal growth rate.

There is clearly considerable complexity in the effect of growth conditions on telomere loss, and measurement stage as well as species life histories are important. For example, in an experimental study in the wild, it has been found that Atlantic salmon *Salmo salar* from the same families have shorter telomere lengths when growing in harsher environments than their siblings growing to the same size in better quality environments (McLennan et al. 2016). However, in contrast, growing fast in good environmental conditions has been associated with shorter juvenile telomere length in the Seychelles warbler *Acrocephalus sechellensis* (van de Crommenacker et al. 2022); telomere lengthening in adulthood has also be reported to occur in this warbler species, so the effect of rapid early growth may not be lifelong (Brown et al. 2022). Telomere lengthening has also been reported in other species. In wild European badgers *Meles meles* for example, it appears that there is a complex pattern of loss and restoration of telomere length over the first three years of life, related to social and environmental conditions. Nonetheless, even when these factors are taken into account, there is still a positive association between badger cub survival and telomere length (van Lieshout et al. 2022).

With respect to reproductive rate, relatively few detailed studies in non-model species have been carried out, which is surprising given that reproductive costs are an important aspect of life history evolution. Telomere loss is potentially a very useful tool to investigate these costs and the evolution of reproductive strategies. However, studying reproductive trade-offs is complex. Individuals differ in the resources they have available, which will influence the extent to which costs can be borne. Accordingly, reproductive effort needs to be experimentally manipulated to uncover physiological costs, deflecting individuals from their optimal investment level (Metcalfe and Monaghan 2013).

Sudyka (2019) carried out a comprehensive examination of the relationship between telomeres and reproductive rates in sexually reproducing species. She found that out of 33 studies, only 7 were experimental, and the majority of these (5/7) supported reproduction-related telomere loss. For correlational studies, the evidence was mixed, as expected. The timescale over which reproductive costs are borne also needs to be taken into account. When zebra finches were allowed to breed from 0 to 5 times a year, telomere loss was greater in the groups that bred compared with the group that did not, irrespective of whether birds bred 1, 3 or five times per year (Heidinger et al. 2012). However, the effect was transient, and no longer evident when telomere length in the same individuals was measured again two years later. Furthermore, the variation in reproductive rate did not affect survival. However, the birds in this study were breeding in captivity, which might have influenced both reproductive costs and extent to which the birds could recover.

## In what ways are telomere dynamics subject to natural selection?

For variation in telomere dynamics to be subject to evolution, the variation needs to be both heritable and have Darwinian fitness consequences. Obviously, the exquisite nature of the telomeric system, preventing as it does the inappropriate triggering of DNA damage responses in species with linear chromosomes, has arisen because of the fitness benefits it confers. Adverse effects are clearly evident when the system malfunctions. But what selection pressures drive the diversity that we see in the detail – in telomere length, and the pattern of loss and restoration, in telomere structure, in the associated proteins and so on? Is management of telomere attrition a high priority that plays a role in life history evolution under natural conditions? Much will depend on the fitness costs of telomere maintenance, about which we know relatively little.

Heritability of telomere length is very important in this context, but difficult to study in the wild. The approach used varies from simple parent offspring regressions to more sophisticated quantitative genetics using the pedigree-based ‘animal model’ (Dugdale and Richardson 2018). The latter is becoming increasingly common in evolutionary ecology, as data from long-term individual-based studies become available. For example, Vedder et al. (2022) in their study of common terns mentioned earlier, used a quantitative genetic approach based on within-individual telomere data collected in adults over a 10-year period from a wild population. Common terns can live over 30 years, and breeders in this population have an average lifespan of 10 years. This population has been studied in detail since 1994 and there are many individually marked individuals whose age and relatedness are known. Thus Vedder et al. had a comprehensive and reliable pedigree to work with. Telomere length was variable but highly heritable, was strongly genetically correlated with lifespan, the rate of telomere loss varied very little among individuals, and environmental effects were limited. Using a similar animal model approach in a similar data set from another long-lived bird in the wild, the jackdaw *Corvus monedula*, in which telomere length and loss was studied based on two measurements taken during the nestling stage, Bauch et al. also found relatively high heritability of telomere length, but more variable shortening rates with low heritability (Bauch et al. 2022). A recent meta-analysis of heritability of telomere length based on 43 studies of 18 vertebrate species reported considerable heterogeneity (Chik et al. 2022). Many factors are involved in generating differences among species and studies. In addition to their different adaptations and evolutionary histories, species might differ also in the degree of variability in the adult and nestling environments. Studies will also vary in the stage over which telomere loss was measured and the method used to measure telomere length. More data are likely to be available from a broader range of species as long-term studies progress. More controlled studies of the fitness consequences of differences in telomere length and loss among and within species are clearly needed.

## Can we use telomere dynamics in a conservation and welfare contexts?

Exposure to environmental stressors is known to increase telomere loss. This has been studied in a wide range of species and environmental circumstances (Haussmann and Marchetto 2010; Monaghan 2014; Angelier et al. 2018). Telomere dynamics are therefore potentially of great interest in conservation and animal welfare contexts, since this could help identify individuals, species and populations threatened by the many challenges posed by rapidly environmental change. Chatelain et al. (2020) conducted a meta-analysis examining the effect of a very broad range of stressful circumstances, both natural and anthropogenic, on telomere length and loss in non-human vertebrates in field and laboratory studies; they found an unequivocally negative effect, consistent across taxa and stressors. Salmon and Burraco (2022) recently also carried out a meta-analysis of the studies investigating how changes in telomere dynamics in wild/wild-derived animals can provide useful indicators of the effect of anthropogenic pollutants (mainly chemical, but also radiation, light and noise) in natural conditions. They concluded that currently, while there is a negative effect overall on telomere length, this is pollutant specific, with the number of studies still being small and the effect weak. They also suggested that the effect could be more marked in endotherms, but the data on ectotherms is very limited. Temperature stress and extreme weather as a consequence of rapid global warming are likely to be faced by many more species, and the pace of change is generally too fast to enable evolutionary adaptation. Some species may be able to migrate to more suitable areas, but many more will face very stressful circumstances which, even if not lethal, will adversely affect their physiology. Such effects may be particularly marked in ectotherms, which are likely to be especially sensitive to thermal stress (Burraco et al. 2020). Changes in telomere loss are likely to provide useful indicators of this stress exposure, and more studies of this are needed. Long lived and large bodied ectotherms do not down-regulate the somatic expression of telomerase to the same extent as many endotherms, and their telomere dynamics are understudied. Using an experimental approach, Zhang et al. (2018) showed that experimental exposure to simulated heat waves appeared to rapidly increase telomere loss in a desert lizard *Phrynocephalus przewalskii.* Dupoue et al. (2022) showed a similar effect in natural populations. They examined telomere length over a 10-year period in three age classes of lizards *Zootoca vivipara* in 22 wild populations facing varying degrees of climate-induced extinction risk. They found that, in declining populations, lizards showed reduced telomere length at all stages, and young lizards in threatened populations inherited already short telomeres. In corals, experimentally induced loss of symbionts is associated with increased telomere loss (Rouan et al. 2022), as is temperature variation (Rouan et al. 2023), indicating that comparisons of telomere length are likely to provide useful indicators of coral reef health. Friesen et al. (2022) recently suggested using indices of thermal sensitivity of ectotherms based on thermal performance curves to aid the study of of this type of stress on ectotherm telomeres.

Effects of high temperatures on telomere length have also been reported in endotherms, being associated with reduced telomere length in nestlings of an endangered bird, the purple-crowned fairy wren *Malurus coronatus* in the wild (Eastwood et al. 2022). Such effects may be stage specific, as seen in zebra finch chicks transitioning during development from ectothermy to endothermy (Ton et al. 2023). In long-lived bats, yearly fluctuation in individual telomere lengths were found to be related to weather factors, suggesting that such changes might provide an index of the level of stress to which individuals are exposed (Foley et al. 2018; Power et al. 2023). It is more likely that telomere dynamics will be adversely affected by exposure to new environmental challenges. Adaptation of aspects of telomere biology to extreme environments, such as low oxygen levels in the underground tunnels of the naked mole-rat *Heterocephalus glaber*, can clearly occur (Augereau et al. 2021). But the degree of within-individual flexibility is unclear and evolutionary adaptation is likely to take many generations. Whether species facing rapid environmental change, such as freshwater fish experiencing low oxygen levels in warmer water, with very little dispersal options, will be able to respond quickly enough remains to be seen.

As well as identifying animals at risk, we need to combine studies at different biological levels to understand processes whereby effective conservation measures might be put in place. Reductions in telomere loss can potentially provide a measure of the extent to which the environment has been improved. For example, as mentioned above McLennan et al. (2016) showed that Atlantic salmon growing fast in a harsh environment, in this case nutrient depleted nutrient upland streams, had greater telomere loss. Importantly, they recently showed that restoration of nutrients in such streams mitigates this adverse effect (McLennan et al. 2022).

With respect to animal welfare, changes in telomere length have a potential application in assessing the welfare of captive animals in a wide variety of situations (Bateson and Poirier 2019), but this has yet to be widely applied.

## Why do we not always see the expected relationships between telomere length and life histories?

Interestingly, despite the very impressive amount of experimental cellular and genetic work establishing causal pathways in humans and model organisms, and demonstration that telomere loss and dysfunction are involved in ageing and age-related disease (Shay and Wright 2019; Lopez-Otin et al. 2023), evolutionary ecologists have sometimes questioned the causal role of telomere attrition in age-related deterioration (Simons 2015). This arises because expected patterns of variation and associations with lifespan are not always seen. However, ageing is not a simple process with a single cause (Lopez-Otin et al. 2023). Furthermore, there are many potential sources of error in telomere measurements, and we need to improve the repeatability and reliability of measurements, especially in evolutionary ecology, where, in addition to the sampling design already mentioned, samples may be of different size and quality, stored for varying amounts of time in sometimes sub-optimal conditions, and analysed by different methods. Also, the proliferative tissues, such as skin and (mostly red) blood cells most often sampled by ecologists, may not always be representative of changes in other body tissues. Some methods of telomere measurement include interstitial repeats of the telomere sequence rather than just the target repeats at the chromosome ends. Since the number of interstitial repeats can vary greatly among species and individuals, this can mask important changes at the chromosome ends. Most methods used by ecologists are relatively simple measures of average telomere length in the sample and sometimes within individual changes in this average length (Nussey et al. 2014). Measurements of the shortest telomeres may well be more informative than averages (Dweck and Maitra 2021) but are as yet little used in evolutionary ecology. The extent to which telomere length and loss are effectively measuring the same thing will depend on the degree of heterogeneity in initial telomere length in the groups(s) studied. The life stage at which telomere length or loss is measured is also relevant, and early life telomere length may be more or less variable due to differences in the stability of the environment at this stage. Age related variation in stem cell recruitment is also likely to occur.

It is also very important to bear in mind that it is telomere *dysfunction* that gives rise to increased frailty and disease. Such dysfunction can be triggered by short telomeres, and average telomere length or the rate of telomere loss is likely to be indicative of increased risk of telomere dysfunction, especially in older individuals. But not always. Telomere dysfunction can occur without changes in telomere length, for example telomere uncapping due to deficiencies in the shelterin proteins involved in blocking the DNA damage response (Chakravarti et al. 2021). Length independent telomere damage can occur in non-mitotic tissues such as cardiomyocytes (Anderson et al. 2019). The importance of such length independent telomere dysfunction may well vary among species and life stages. An important area where collaboration between biomedical scientists and evolutionary ecologists would be particularly fruitful is in developing assays for telomere dysfunction that could be used in non-model species and applied in wild populations.

But there are fundamental reasons why we should not expect to see the same pattern across all species, which cannot be addressed by improvements in sampling design and methodology. Evolutionary ecologists now often use meta-analyses to look for general patterns across a broad range of species. In the context of telomere dynamics, this type of analysis is particularly suited to studies investigating the adverse effects of particular environmental factors, such as when the question is - does exposure to a particular type of anthropogenically generated chemical, which is not part of the ‘natural’ environment to which the animals are adapted, increase telomere loss? It is not surprising therefore that we see the most consistent effects across species when we examine the effect of pollutants or temperature. Meta-analyses are much less suited in answering questions such as, does variation in telomere length (or loss) predict lifespan? Different species are likely to have evolved different solutions to the optimal management of telomere dysfunction. In some species, telomere loss will ‘matter’ in fitness terms, while in others it will not. When the overall effect size is weak, or effects are only apparent in some species but not in others, the inconsistency in results should not be taken as evidence that changes in telomere dynamics are not informative about ageing or life history evolution. Rather, it should help us identify when telomere length or loss does and does not ‘matter’. Better still, we might construct hypotheses to this effect. In species with high mortality risk, where lifespan is likely to be very short for most individuals due to ephemeral resources or high predation, cells are unlikely to undergo sufficient divisions for telomere shortening to 'matter' in evolutionary terms. In this case, variation in telomere loss maynot be related to fitness traits. Evolutionary ecologists generally celebrate variation, since understanding why traits differ within and among species is central to our discipline. We should expect evolution to have tailored ancestral molecular, morphological and physiological systems to species specific requirements. We must therefore expect differences in the extent to which telomere length or loss map on to differences in longevity and other life history traits.

We still know little about why telomere length varies greatly among species, or about the costs associated with having long telomeres. Does this slow the cell cycle and might the costs and benefits of this vary? Additionally, much depends on the costs of telomere maintenance. Studying a diversity of species will not only help us understand fundamental telomere biology better, but potentially also provide novel insights into the ageing process and age-related diseases (Quesada et al. 2019). To do so, we need to build more inter-disciplinary bridges.
